# Genome Sequence of a Heterotrophic Nitrifier and Aerobic Denitrifier, Paracoccus denitrificans Strain ISTOD1, Isolated from Wastewater

**DOI:** 10.1128/genomeA.00210-18

**Published:** 2018-04-12

**Authors:** Kristina Medhi, Arti Mishra, Indu Shekhar Thakur

**Affiliations:** a School of Environmental Sciences, Jawaharlal Nehru University, New Delhi, India

## Abstract

We report here the draft genome sequence of Paracoccus denitrificans strain ISTOD1 of 4.9 Mb, isolated from wastewater. It has been identified as a heterotrophic nitrifying and aerobic denitrifying bacterium. Genomic analysis revealed genes related to nitrogen and phosphorus removal, showing that the strain holds potential for bioremediation and biorefinery uses.

## GENOME ANNOUNCEMENT

Nitrogen (N) and phosphorus (P) are vital components of biogeochemical cycles and are considered essential nutrients. However, overabundance of these nutrients in the present-day environment is causing deterioration of the water quality and ultimately affecting human health. Domestic and industrial sewage contributes 50% of the nutrient overload received by the water bodies (1). Bacteria play a very important role in wastewater treatment (2, 3). Paracoccus denitrificans is well known for its capability to denitrify nitrogen and remove phosphorus (4, 5). A heterotrophic nitrifying-aerobic denitrifying bacterium, Paracoccus denitrificans strain ISTOD1, was isolated from wastewater samples from the Okhla Sewage Treatment Plant, New Delhi, India. The nitrogen removal rate and kinetics with this strain were investigated under both aerobic and anaerobic conditions (2). The whole-genome sequencing carried out might provide insight into how to obtain detailed information on its enzymes and the pathways required to build a sustainable biorefinery concept.

The draft genome sequencing of Paracoccus denitrificans strain ISTOD1 was performed on the Illumina NextSeq 500 sequencing system with a paired-end library. A total of 9,457,606 paired-end reads were generated, from which, after quality trimming, error correction, and filtering, 8,608,442 (96.38%) high-quality paired-end reads were retained. Sequence processing and genome assembly were performed using SPAdes version 3.10.1 and Velvet (6). The primary assembled contigs were subjected to scaffolding and gap filling using BaseClear SSPACE standard (7) and BaseClear Gap Filler (8), respectively. The assembly resulted in 63 scaffolds of 4,902,260 bp, with an *N*_50_ scaffold size of 329,769 bp. The maximum and minimum scaffold lengths were 465,696 bp and 144 bp, respectively. The final genome assembly of Paracoccus denitrificans strain ISTOD1 contained 4,902,260 bp, as assessed by a k-mer counting tool, with a G+C content of 67%. The assembly resulted in a median coverage of 578×. Genome annotation using Prokka (9) revealed 4,697 protein-coding sequences, 52 tRNAs, 3 rRNAs, 1 transfer-messenger RNA (tmRNA), and 32 miscellaneous RNAs. The genes involved in pathways were predicted by using the KEGG Automatic Annotation Server (KAAS) (10). Based on the annotation, CVTree (11) was used to create a phylogenetic tree using the Proteobacteria lineage, which consists of 1,043 genomes, including the Paracoccus denitrificans genome.

The strain ISTOD1 genome harbored a cluster of genes involved in denitrification (*nar, nir, nor,* and *nos*), and it revealed putative ammonia monooxygenase genes responsible for the nitrification process. Phosphorus removal genes (*ppk* and *ppx*) were also identified. The genome is equipped with genes responsible for carbon metabolism, antibiotic biosynthesis, and degradation of aromatic compounds, like polyaromatic hydrocarbons (PAHs) and nitrocompounds. Genes related to RuBisCo (*cbbS* and *cbbL*) and polyhydroxyalkanoate (PHA) synthesis reveal its application in CO_2_ sequestration, while some genes conferring resistance to arsenic, bicyclomycin, organic hydroperoxide, and fosmidomycin were also found. The genome is also enriched with genes having capsular polysaccharide biosynthesis and export, whereas some uncharacterized proteins are involved in exopolysaccharide (EPS) biosynthesis. Strain ISTOD1 has versatile traits, and its sequencing will help us to understand its role in the bioremediation and applicability in biovalorization.

### Accession number(s).

The genome sequence discussed here was submitted to DDBJ/EMBL/GenBank under the accession number PPGA00000000.
